# Effect of various supplements on productive performance of honey bees, in the south Wollo Zone, Ethiopia

**DOI:** 10.1371/journal.pone.0303579

**Published:** 2024-05-29

**Authors:** Wubalem Alebachew Amera, Berhan Tamir Mersso, Tadesse Amare Sisay, Amssalu Bezabeh Arega, Abiyu Tadele Alene

**Affiliations:** 1 Department of Animal Science, Injibara University, Injibara, Ethiopia; 2 Department of Animal Production Studies, Addis Ababa University, Addis Ababa, Ethiopia; 3 Department of Animal Science, Wollo University, Dese, Ethiopia; 4 Holeta Bee Research Center, Oromia Institute of Agricultural Research, Oromia, Ethiopia; 5 College of Agriculture and Natural Resources, Bonga University, Ethiopia; Institute of Apicultural Research, CHINA

## Abstract

The productivity and well-being of honey bee colonies are greatly influenced by the nutrients present in the hives. A study was conducted to evaluate different supplemental feeds on honey bee productive performance during dearth periods. Thirty colonies were grouped into five (four treatment groups and one control group) and each group contained three sub-groups (2 weak, 2 strong, and 2 very strong). Control groups were not given any supplementation. Treatment diets were T1 (50% sugar syrup + 14% roasted barley powder (beso) + 36% roasted spiced pea powder (Shiro)), T2 (50% powder sugar + 14% white sorghum powder + 36% bakery yeast, T3 (50% powder sugar + 14% white sorghum powder + 36% skimmed milk powder), T4 (50% sugar syrup with infusion of stinging nettle and 1% kerefa + 50% white sorghum powder). Feed was given on the entrance sides. The performance of experimental colonies was measured every 21 days in two phases during the dry season (from 3_2_2021 to 27_4_2021) and the rainy season (from 28–7_2021 to 1_10_2021). Feed intake, space (cm2) of pollen, nectar, and honey in the comb were measured using a frame-sized transparent grid meter. The study revealed significant differences (p<0.0001) in all measured parameters among the various treatments. The diet provided by T4 showed the highest levels of crude protein (18.15%) and carbohydrates (92.15%), whereas the diet presented by T3 had the lowest crude protein content (6.66%) and the diet offered by T1 had the lowest carbohydrate content (61.91%). In general, colonies that received T4 showcased superior performance compared to others. They exhibited a feed intake of 98.3%, a nectar area of 54.3 cm2, a pollen area of 68.7 cm2, a honey area of 311.2 cm2, and a honey yield of 7 kg. Consequently, their net profit amounted to 51.54 USD. On the other hand, the colonies that received T1 had the lowest performance indicators. They demonstrated a feed intake of only 54.7%, a nectar area of 37.6 cm2, a pollen area of 48.8 cm2, a honey area of 254.3 cm2, a honey yield of 2.8 kg, and a net profit of 18.81 USD. The significance of this study was to enable the beekeepers in realizing the effects of feed supplements on the productivity and profitability of honeybee colonies.

## 1. Introduction

The well-being, lifespan, and progress of honey bee colonies rely heavily on the presence and caliber of nutrients within their hives. To survive, reproduce, and withstand stress, bees require access to nectar and pollen that encompass a mix of carbohydrates, proteins, lipids, and micronutrients. Typically, honey bees acquire carbohydrates from nectar and protein from pollen, ensuring they have a balanced diet. However, various factors like climate change, agricultural intensification, alterations to landscapes, and atmospheric pollutants can impact the abundance and productivity of plant populations [1–4]. According to research conducted by Ghosh *et al*. [5], it was discovered that colonies of bees have varying preferences for pollen based on its protein content. Bees tend to favor floral sources that offer higher nutritional value. Furthermore, Radev *et al*. [6] and Kumar and Agrawal [7] emphasize that plant flora changes throughout different seasons, impacting colony development [6]. Further studies conducted by Brodschneider and Crailsheim [8], Radev *et al*. [6] as well as Zheng *et al*. [9] indicate a definitive link between the nutritional value of pollen and the overall development, reproduction, and productivity of bee colonies.

In the field of beekeeping, pollen, and honey-based supplementary feeding are employed to combat hunger and promote greater population growth within bee colonies during the spring season. This practice primarily focuses on facilitating brood formation among young worker bees and supporting the proliferation of queens and drones. The objective behind these efforts is to ensure the overall health of the colonies, reducing the risk of bee loss resulting from the application of agricultural chemicals in plant production [10–12]. It is crucial to note that the longevity of honey bees is significantly influenced by the composition and proportion of nutrients they receive [13]. According to research findings by Şahin *et al*. [14], floral resources undergo significant reductions throughout the year due to seasonal and climatic changes such as extreme temperatures, precipitation, and hail. This scarcity of natural flora has a direct impact on the egg-laying capacity of queen bees, leading to a decrease in population levels within bee colonies. Insufficient nutrition contributes to a lower survival rate among individuals, resulting in premature mortality during the larval stage. Furthermore, malnutrition makes the colony more vulnerable to diseases and prompts some individuals to abandon the colony [3]. Numerous countries have conducted feeding studies on honey bees and bumblebees, as evidenced by research studies in various nations [12, 15–18]. Furthermore, scientific models have been devised to explore the impact of these nutrients on colony growth, providing insights into the future outlook for bee colonies [19]. During the pine honey season in autumn, when pollen sources are scarce, colonies tend to weaken following the honey harvest. Therefore, it becomes crucial to implement appropriate feeding strategies for wintering to overcome this challenge [20]. However, the practice of protein source feeding is not followed because Beekeepers in Ethiopia mostly feed their honeybee colonies with sugar syrup, *beso* (roasted and grounded barley flour), *Shiro* (roasted spiced pulses flour), and honey [21–24]. However, there is no such finding about the effects of these feeds on colony performances. This study aimed to evaluate the effects of different supplementations on honey bee productive performance and determine the economic feasibility of the diets.

## 2. Materials and methods

### 2.1. Description of the study area

This study was conducted in Dessie Zuria which is in the district of the south Wollo Zone and located between 6°20’ to 8°0’ North latitude and 35`1°40’ to 37°20’ East longitude in Amhara Region, Ethiopia. It has an area of 937.32 km^2^, located about 401 km North of Addis Ababa. Dessie is the administrative capital of the district and Dessie Zuria is bordered on the South by Wereillu and Albuko, on the Southwest by Legambo, on the Northwest by Tenta, on the North by Kutaber, on the Northeast by Tehulederie, on the East by Kalu district. Dessie Zuria district has the lowest temperature ranging from 10°C to 16°C, because of the high altitude. In these districts, the majority of the farmer’s practice rain-fed agriculture but rainfall is either erratic or low in most times. Many areas in Dessie Zuria receive high rainfall ranging between 1200 and 1400 mm. Dessie Zuria district has a total population of 157,679 of whom 77,626 are men and 80,053 women and all are rural inhabitants [25].

### 2.2. Experimental design and treatments

#### 2.2.1. Apiary site preparation

To prevent robbery bees that share feeds, the trials were conducted at an optimum distance away from the others. Thirty honey bee colonies (*A*. *mellifera monticolla*) were used in this experiment. Before the experiment, these colonies were divided into three groups weak, strong, and very strong based on their strength through colony equalization. Then these groups were randomly grouped into each treatment diet, by using a randomized complete block design (RCBD) with six replications (six colonies per treatment (each treatment group contains 2 weak, 2 strong, and 2 very strong colonies)).

#### 2.2.2. Colonies management

**Fig 1:**
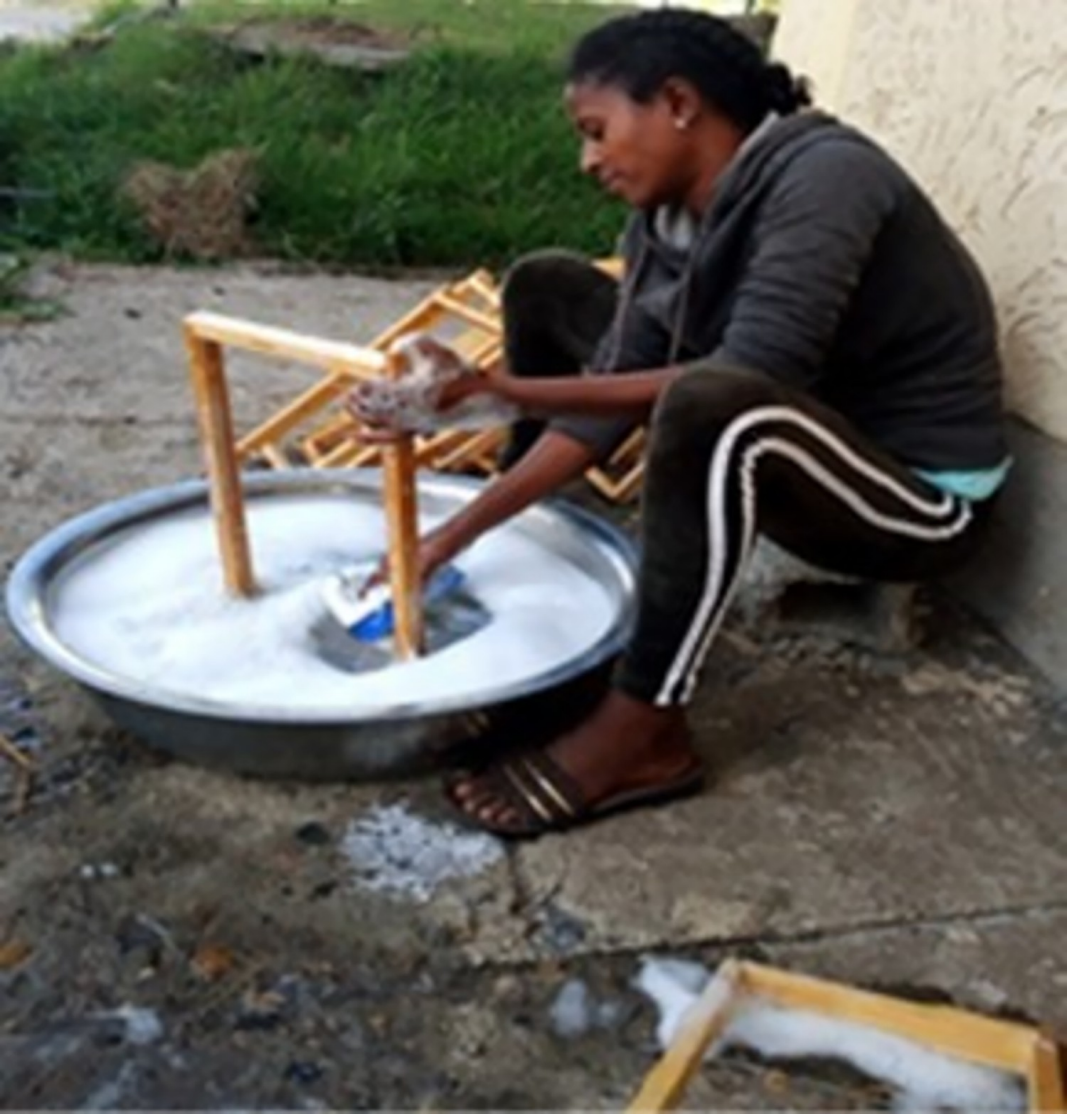
Frame washing.

Before transferring, artificial honey comb (S1 Fig) was prepared, fixed to the hives, both hives and frames were well washed, disinfected, and fumigated to avoid pests but attract honeybee colonies (Fig 1). Until transferring, traditional hive colonies were given sugar syrup and other locally available supplementation.

#### 2.2.3. Diet preparation and administration

Stinging nettle infusion was prepared from nettle plant leaf powder (meal) (100 g nettle leaf powder in 1000 mL hot water). The infusion was used for preparing the syrup (S2 Fig). Sorghum and Barly powders, *kerefa* (*Cinnamomum Zelanicum*), and sugar powder are prepared by grinding, and *Shiro* is also prepared by roasted spiced pea flour. Skimmed milk powder and bakery yeast, were purchased from the market directly. The sugar syrup was prepared with a mixture of sugar and water at a 1:1 ratio. Syrup for Diet 4 was made by infusion of Nettle. All dietary treatments were formulated as shown below. The feeds were rationed based on the energy and protein requirements of honey bees.

**T1** = 50% sugar syrup + 14% roasted barley powder + 36% roasted spiced pea powder (*Shiro*).

**T2 =** 50% powder sugar + 14% white sorghum powder + 36% bakery yeast

**T3 =** 50% powder sugar + 14% white sorghum powder + 36% skimmed milk powder

**T4:** 50% sugar syrup with the infusion of stinging nettle and 1% *kerefa* + 50% white sorghum powder.

**C:** Control groups were not given any supplements.

**Fig 2:**
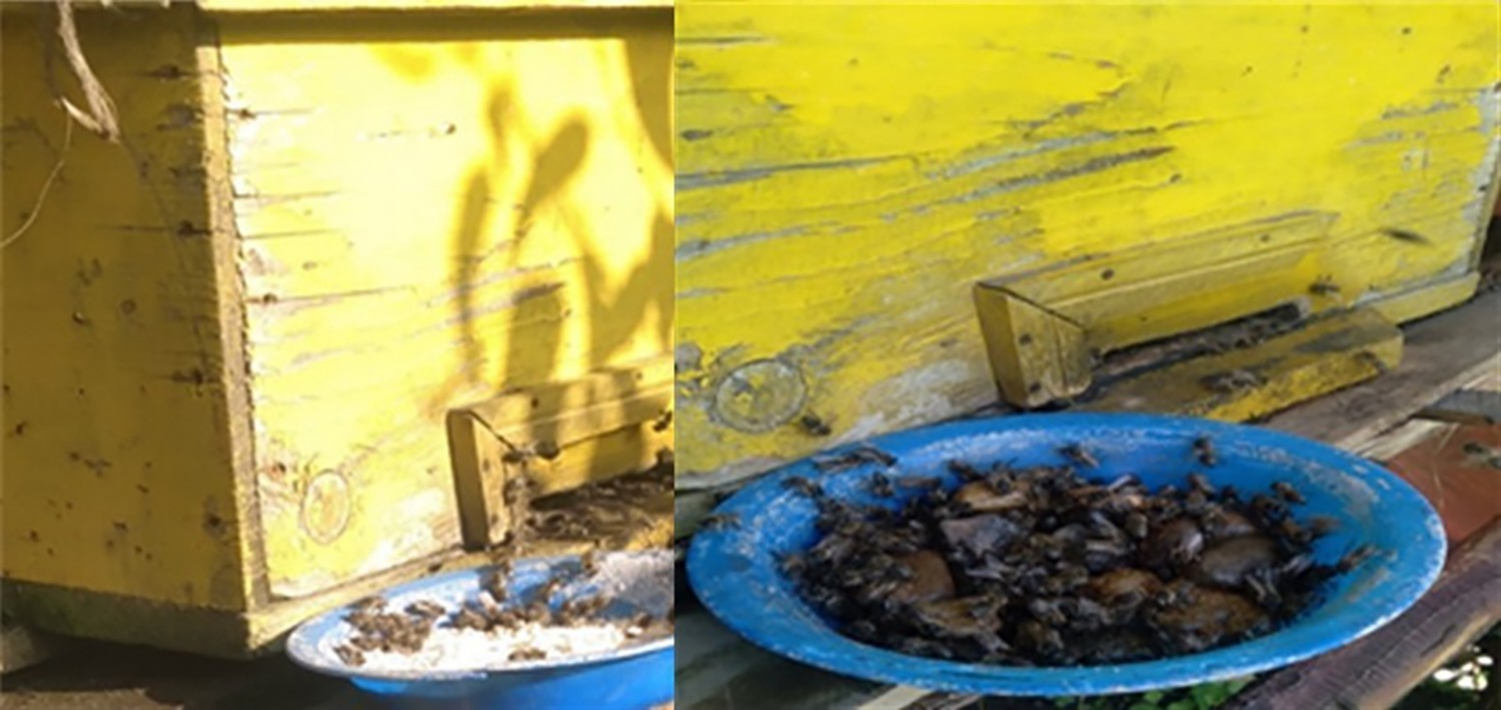
Feed supplementation (bakery yeast content of feed (left) and syrup of nettle infusion (right)).

To prevent other animals from consuming the feed, it was monitored throughout the day placed on the entrance (Fig 2) at 5:00 in the morning, and taken down at 7:20 at night. Depending on their consumption level, the feed was changed every week with freshly produced feed. Depending on their strength, a weekly amount of 100, 150, or 200 g of feed was given, and their feed intake was measured. To safeguard the freshness of the feeds, any color change of the water and feed in troughs were monitored carefully and the color of troughs was considered the color preference of honey bees.

### 2.3. Performance parameter measurements

**Fig 3:**
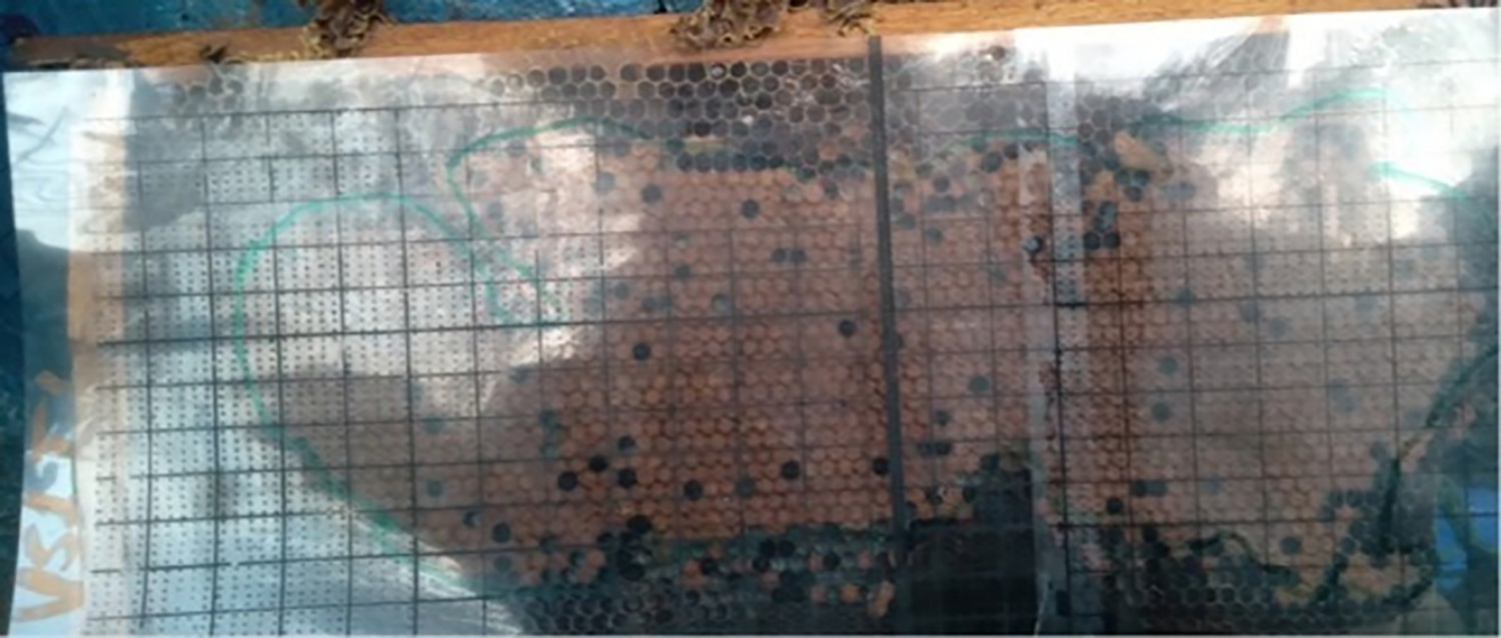
Performance measurements.

With a 1 cm x 1 cm grid of clear plastic connected to the frame, the amount of space (in cm^**2**^) occupied by pollen and nectar was evaluated [26, 27]. This grid frame was used to estimate the storage of pollen and nectar by placing it on top of the comb’s surface (Fig 3). These data were collected during the dearth period from 3 Feb to 27 April/2021 and the wet season from 28 July to 1 Oct/2021. To reduce errors, data relating to pollen and nectar was gathered from all experimental colony frames. Honey data was gathered when the honey was harvested. Weight differences between frames containing honey before and after honey extraction were recorded as the honey harvest/yield. Honey store also determined.

Initially, all treatments had an equal amount of the following parameters:

Nectar area = 30 cm^2^   egg area = 121.7 cm^2^

Pollen area = 45 cm^2^   larvae area = 270 cm^2^

Honey area = 300 cm^2^   pupa area = 370 cm^2^

Colony strength = 4.2 frames covered by honey bees

### 2.4. Partial budget analysis

Partial budget analysis was calculated based on the total cost of feed preparation which is the cost of feed and labor cost and based on return, income generated from honey. The net profit was calculated by deducting the total cost of feeds and labor from the income generated.

### 2.5. Laboratory analysis

At the Holeta Agricultural Research Center, a comprehensive analysis of various nutritional components was carried out, encompassing crude protein (CP), carbohydrate (CH), crude fat (CF), crude fiber (CFi), organic matter (OM), moisture (moist.), dry matter (DM), and ash. To measure the dry matter content of a 1 g feed sample, it was exposed to a 105°C oven for a duration of 24 hours. The Kjeldahl method of nitrogen analysis was employed to determine the crude protein content in the samples, while the protein content was computed using the formula N*6.25. To determine the dry matter and crude protein content, standard analysis procedures were adhered to.

To analyze the fiber fractions of the samples, a range of solutions was prepared. These included an acid detergent solution, used for acid detergent fiber analysis, potassium permanganate and demineralized solutions, used for lignin analysis, and a neutral detergent solution for neutral detergent fiber analysis. Both the neutral detergent fiber and acid detergent fiber were analyzed using a similar procedure, with the only difference being the solutions used. To begin, a solution was prepared. Next, a sample weighing 0.5g was combined with 50 mL of the respective solution and boiled for one hour. The resulting residues were then filtered and subjected to drying in an oven at 105°C for a period of 24 hours.

### 2.6. Statistical analysis

The performance differences were tested using the analysis of Variance (ANOVA) procedure of the SAS general linear model (GLM). The least significant difference (LSD) at a 5% significant level was used for comparisons of means.

The productive performance of the response variables was subjected to different treatment diets.

Model

Yij=μ+ti+eij

Where, Yij = represents the j observation in the i^th^ treatment group; μ = overall mean;

ti = treatment effect (T1, T2, T3 & T4); and eij = random error [28].

## 3. Results

The nutritional composition of experimental diets is demonstrated in Table 1. Among the different treatments (T_1_, T_2_, T_3_, and T_4_), treatment 4 (T_4_) displayed the highest content of crude protein (CP) at 18.15%. In contrast, treatment 3 (T_3_) exhibited a lower value of CP at 6.66%. When it comes to crude fat (CF), treatment 1 (T_1_) had the highest amount at 5.96%. On the other hand, treatment 2 (T_2_) had the lowest value of CF among all the diets, measuring at 1.85%. In terms of carbohydrate content, Treatment 4 (T_4_) had the highest percentage at 92.15%, followed by T_3_ at 83.95%, T_2_ at 75.06%, and T_1_ at 61.91%. Looking at crude fiber (CFi), T_3_ had the lowest value at 4.65%, while T_1_ had the highest at 6.76%.

**Table 1:** Nutritional composition of experimental feeds (Mean ± SE).

Nutritional composition	Treatments				
	T_1_	T_2_	T_3_	T_4_	P-value
**CP (%)**	16.37^b^±0.02	14.77^c^±0.02	6.66^d^±0.01	18.15^a^±5.91	0.001
**CF (%)**	5.96^a^±0.02	1.85^b^±0.01	4.45^a^±0.03	5.75^a^±0.56	0.001
**CH (%)**	61.91^d^±0.01	75.06^c^±0.01	83.95^b^±0.01	92.15^a^±1.46	0.001
**CFi (%)**	6.76^a^±0.01	6.29^a^±0.02	4.65^a^±0.01	6.65^a^±1.30	0.001
**Ash (%)**	7.51^a^±0.05	2.26^b^±0.07	1.68^b^±0.05	8.7^a^ ±4.45	0.001
**OM (%)**	92.49^b^±0.05	97.75^a^±0.07	98.33^a^±0.05	98.3^a^±0.46	0.001
**Moist. (%)**	8.23^b^±0.01	5.93^c^±0.05	3.26^d^±0.02	11.25^a^±0.25	0.001
**DM (%)**	91.77^d^±0.01	94.07^c^±0.05	96.74^b^±0.02	98.8^a^ ±0.29	0.001

CP: Crude Protein; CF: Crude Fat; CH: Carbohydrate; CFi: Crude Fiber; OM: Organic Matter; Moist.: Moisture; DM: Dry Matter; **T1:** 50% sugar syrup + 14% roasted barley powder (*beso*) + 36% roasted spiced pea powder (*Shiro*); **T2:** 50% powder sugar + 14% white sorghum powder + 36% bakery yeast; T**3:**50% powder sugar + 14% white sorghum powder + 36% skimmed milk powder **T4:** 50% sugar syrup with infusion of stinging nettle and 1% *kerefa* + 50% white sorghum powder; C: not given any supplementation. SE, standard error; ^a-d^ means that different superscripts are significantly different.

Concerning ash content, T_4_ recorded the highest value at 8.7%, whereas T_3_ had the lowest value at 1.68%. Both T_4_ (98.3%) and T_3_ (98.33%) contained the highest organic matter (OM) content, followed by T_2_ at 97.75% and T_1_ at 92.49%. The moisture (Moist.) content ranked highest in T_4_ at 11.25%, followed by T_1_ at 8.23%, T_2_ at 5.93%, and T_3_ at 3.26%. Treatment 4 (T_4_) exhibited the highest dry matter (DM) content, reaching an impressive 98.8%. Following closely behind, T_3_ showcased a DM content of 96.74%, and T_2_ recorded 94.07%. In contrast, T_1_ displayed the lowest DM content, measuring a minimum value of 91.77%. These findings indicate statistically significant differences in nutritional compositions among all treatments *(p < 0*.*05*).

Table 2 shows the feed intake of honey bees with different supplementations. All experimental colonies, except for the control group, were provided with an equal amount of feed. The control group did not receive any supplements. The intake of supplement feeds showed significant variation (*p < 0*.*05*). Experimental honeybee colonies consumed approximately 98% of the supplement feeds provided under T_4_ and T_2_, whereas only 54% of T_1_ and 60% of T_3_ supplements were consumed by experimental honeybee colonies. The experimental findings demonstrate that the intake of supplements was noticeably higher in treatments two (T_2_) and four (T_4_) compared to the other treatments (*p < 0*.*05*). Statistical analysis indicated no significant variation in consumption between T_4_ and T_2_, as well as T_1_ and T_3_ (*p > 0*.*05*). These results indicate that honeybee colonies find the feeds provided under treatments 4 (T_4_) and 2 (T_2_) more appealing than the other feeds.

**Table 2:** Effects of different supplemental diets on feed intake of honey bee colonies (Mean ± SE).

Parameters	T_1_	T_2_	T_3_	T_4_	C	P value
**FO(g)**	150.0^a^±3.5	150.0^a^±3.5	150.0^a^±3.5	150.0^a^±3.5	0^b^±0	0.001
**FR(g)**	68.2^a^±1.9	1.5^b^±0.1	59.5^a^±1.7	1.3^b^±0.1	0^c^±0	0.001
**FI(g)**	81.8^b^ ±1.9	148.5^a^±3.5	90.5^b^±2.3	148.7^a^±3.5	0^c^±0	0.001
**Perc.(%)**	54.7^b^±0.6	98.8^a^±0.1	60.4^b^±0.6	98.3^a^±0.7	0^c^±0	0.001

FO: Feed Offered; FR: Feed Refusal; FI: Feed Intake; Perc: Percent; **T1:** 50% sugar syrup + 14% roasted barley powder (*beso*) + 36% roasted spiced pea powder (*Shiro*); **T2:** 50% powder sugar + 14% white sorghum powder + 36% bakery yeast; T**3:**50% powder sugar + 14% white sorghum powder + 36% skimmed milk powder **T4:** 50% sugar syrup with infusion of stinging nettle and 1% *kerefa* + 50% white sorghum powder; C: not given any supplementation. SE, standard error; ^a-c^ means that different superscripts are significantly different

The experiment’s overall colony productivity is summarized in Table 3. In terms of the nectar area (S1 Table), T_4_ (54.3 cm^2^) gathered a greater amount of nectar compared to T_2_ (50.1 cm^2^), T_3_ (40.7 cm^2^), or T_1_ (37.6 cm^2^). However, the statistical analysis revealed that the difference between T_4_ and T_2_ was not significant (*p > 0*.*05*). On the other hand, there were no significant differences detected between T_1_ and T_3_. The control colonies registered the smallest value of 16.4 cm^2^, which was significantly lower (*p < 0*.*05*) compared to all other experimental colonies. While there was a minimal difference in pollen area measurement (S2 Table) between T_4_ (68.7 cm^2^) and T_2_ (64.6 cm^2^), T_4_ consistently maintained the highest value. The control colonies exhibited the lowest values of 21.6 cm2 and displayed significant differences (*p < 0*.*05*) compared to the other experimental colonies. There was no noticeable difference observed between T_1_ (48.8 cm^2^) and T_3_ (53.3 cm^2^), as well as between T_2_ and T_3_.

**Table 3:** Cumulative colony productive performance at the end of the experiment (Mean ± SE).

Parameters	T_1_	T_2_	T_3_	T_4_	C	P value
NA (cm^2^)	37.6^b^±1.3	50.1^a^±2.6	40.7^b^±1.8	54.3^a^±3.2	16.4^c^±1.3	0.001
POA (cm^2^)	48.8^c^±2.5	64.6^ba^±4.3	53.3^bc^±2.8	68.7^a^±4.5	21.6^d^±1.9	0.001
HA (cm^2^)	254.3^b^±10.5	295.6^ba^±10.9	262.3^b^±10.2	311.2^a^± 12.8	103.9^c^±12.8	0.001
**HY (kg)**	2.8^b^±0.2	6.0^a^±0.7	3.8^b^±0.6	7.0^a^±0.7	0.7^c^±0.1	0.001

**T1:** 50% sugar syrup + 14% roasted barley powder (beso) + 36% roasted spiced pea powder (Shiro); **T2:** 50% powder sugar + 14% white sorghum powder + 36% bakery yeast; T**3:**50% powder sugar + 14% white sorghum powder + 36% skimmed milk powder **T4:** 50% sugar syrup with infusion of stinging nettle and 1% kerefa + 50% white sorghum powder; C: not given any supplementation**. NA:** Nectar Area; **POA:** Pollen Area; **HA:** Honey Area; **HY:** Honey Yield. SE, standard error; ^a-d^ means that different superscripts are significantly different.

The experimental diets had an impact on the honey area as well. Among the colonies, those that received T_4_ (311.2 cm^2^) showed the highest value, while T_2_ (295.6 cm^2^) did not significantly differ (*p > 0*.*05*) from T_4_. While T_3_ measured 262.3 cm^2^ compared to T_1_’s 254.3 cm^2^, there was no statistically significant difference between T_3_, T_1_, and T_2_ (*p > 0*.*05*). In the comparison of control colonies (103.9 cm^2^), it was observed that their honey area (S3 Table) was smaller than all experimental colonies, and this difference was found to be statistically significant. Among the experimental colonies, T_4_ had the highest honey yield of 7 kg, followed by T_2_ with 6 kg. However, there was no statistically significant difference between T_4_ and T_2_. T_3_ (3.8 kg) and T_1_ (2.8 kg) displayed higher honey yields (S4 Table) compared to the control groups (0.7 kg). Moreover, a statistically insignificant difference (*p > 0*.*05*) was observed between the honey yields of T_1_ and T_3_.

Table 4 presents the profitability of different experimental diets. Among them, T_4_ showed the lowest cost of feed, with a value of 1.30 USD, whereas T_3_ had the highest cost at 8.34 USD. There was no significant difference (*p > 0*.*05*) between T_1_ (2.16 USD) and T_2_ (3.15 USD) or T_4_ in terms of feed cost. It is worth noting that the labor cost remained unchanged across all treatments. In terms of gross expenditure, T_3_ had the highest value of 8.37 USD, followed by T_2_ (3.17 USD), T_1_ (2.18 USD), and T_4_ (1.32 USD). However, there was no statistically significant difference (*p > 0*.*05*) between T_1_ and T_2_ or T_1_ and T_4_.

**Table 4:** Profitability of experimental diets in USD (Mean ± SE).

Parameters (USD)	T_1_	T_2_	T_3_	T_4_	C	P value
**CoF**	2.16^cb^±0.18	3.15^b^±0.22	8.34^a^±0.55	1.30^c^±0.109	0^d^±0	0.001
**CoL**	0.02^a^±0.01	0.02^a^±0.01	0.02^a^±0.01	0.02^a^±0.01	0^a^±0	0.702
**GE**	2.18^cb^±0.17	3.17^b^±0.21	8.37^a^±0.54	1.32^c^±0.10	0^d^±0	0.001
**P_of_h/kg**	7.488±0.133	7.488±0.133	7.488±0.133	7.488±0.133	7.488±0.133	1.000
**GR**	21.00^dc^±1.55	45.52^ba^±5.42	29.07^bc^±4.52	52.86^a^±5.73	4.845^d^±0.926	0.001
**NP**	18.82^b^±1.43	42.35^a^±5.30	20.71^b^±4.12	51.54^a^±5.66	4.845^c^±0.926	0.001

COF: Cost of Feed; COL: Cost of Labor; GE: Gross expenditure; P of h/kg: price of honey per kg; GR: Gross Return; NP: Net Profit; Kg: Killo gram; **T1:** 50% sugar syrup + 14% roasted barley powder (*beso*) + 36% roasted spiced pea powder (*Shiro*); **T2:** 50% powder sugar + 14% white sorghum powder + 36% bakery yeast; T**3:**50% powder sugar + 14% white sorghum powder + 36% skimmed milk powder **T4:** 50% sugar syrup with infusion of stinging nettle and 1% *kerefa* + 50% white sorghum powder; C: not given any supplementation. SE, standard error; ^a-d^ means with different superscripts are significantly different.

The honey treatment colonies were all charged an equal amount of 7.488 USD. Among the treatments, T_4_ (52.86 USD) had the highest impact on gross return, followed by T_2_ (45.52 USD). However, there was no statistically significant difference between T_4_ and T_2_ (*p > 0*.*05*). The control colonies, on the other hand, had a lower value of 4.847 USD compared to the other colonies. The difference between the control colonies and T_1_ was statistically insignificant (*p > 0*.*05*). There were no significant differences (*p > 0*.*05*) observed among T_1_ (21 USD), T_2_, and T_3_ (29.05 USD). However, the colonies treated with T_4_ (51.54 USD) showed the highest net profit compared to those treated with T_2_ (42.35 USD), T_3_ (20.71 USD), or T_1_ (18.82 USD), as well as the control colonies which had the lowest net profit (4.847 USD). Statistical analysis revealed no significant difference (*p > 0*.*05*) between T_1_ and T_3_, as well as between T_2_ and T_4_.

## 4. Discussion

The research studies conducted by [29–31] have confirmed the nutritional composition of stinging nettle in T4. Additionally, the nutritional composition of white sorghum in T_2_, T_3_, and T_4_ is supported by the findings of [32–37]. According to various studies Monika *et al*. [38] and Agrawal *et al*. [39], it has been confirmed that the yeast in T_2_ exhibits a higher nutritional composition. Similarly, the favorable nutritional composition of Skimmed milk found in T_3_ aligns with the discoveries made by [7, 40]. Paray *et al*. [41] Conducted research on powder honey bee pollen supplementation using Skimmed milk and soya bean flour.

For honey bee colonies to thrive in situations where natural nectar is scarce, it is crucial to provide them with supplemental sugar feed. The type of sugar feed plays a significant role in influencing the development and well-being of bee colonies, ultimately enhancing their resistance to various factors [42, 43]. Recent studies by Mohammed *et al*. [32], Agarwal *et al*. [39], and Al Mărghitaş *et al*. [44] have indicated that white sorghum powder and nettle infusion syrup, respectively, exhibit greater appeal to honey bees. According to the research conducted by Haleem *et al*. [45], El-Wahab *et al*. [40], and Agarwal *et al*. [39], it has been found that honey bees are attracted to yeast and skimmed milk powder. Additionally, the study carried out by Ricigliano *et al*. [46] indicated that sugar-negative feed is not as preferred by honey bees compared to sugar-positive alternatives. This finding supports the lower intake of feed observed in T_1_ during the current study.

The study conducted by Ullah *et al*. [47] revealed that the highest weekly ingestion recorded was (74.34 g), which was comparatively lower than the findings of the present study (98.2%). Conversely, the results obtained by Mahesh *et al*. [48], who explored different supplement combinations, indicated a maximum net consumption of (67.85 g), which was also lower than the feed intake observed in the current study. Furthermore, Islam *et al*. [49], reported that honey bees consumed a maximum of 71.90 g of food per week, which is lower than the findings of the present study. On the other hand, El-Wahab *et al*. [40], documented a higher result in terms of maximum total feed intake (100%) compared to the current findings.

In colonies that were provided with T_4_, the nectar area reached its maximum size, measuring 54.3 cm^2^. However, the results obtained in our study were comparatively lower than those reported by Hunde [50]. Their research, which involved supplementing the diet with soya beans, revealed a significantly larger nectar area of 258.9 cm^2^. The colonies that were fed with T_4_ and T_2_ exhibited higher feed intake and benefited from a nutritional composition that contributed to a larger brood area and increased colony strength. The correlation between these factors has been extensively studied and supported by various researchers. For instance, Rodney and Purdy [51] discovered a strong link between nutrition and the quantity of nectar in bee hives. They found that the availability of nectar is influenced by the nutritional factors affecting honey bee colonies. Additionally, Chaand *et al*. [52] highlighted that the strength of a colony has a direct impact on the presence of pollen, nectar, and honey reserves. Another study conducted by Sihag and Kaur [53] further reinforced these findings by establishing a positive association between pollen and nectar stores, as well as brood and colony strength.

In the current study, there was a slight reduction observed in the nectar area compared to its initial size. This finding aligns with the research conducted by Huang [54] and Amro *et al*. [55], who found that honey bees tend to show a preference for natural pollen and nectar over supplemented and substitute diets. In this study, colonies that received T_4_ exhibited a relatively higher honey area of 311.2 cm^2^. However, it should be noted that this value is lower compared to the honey areas reported by Sihag and Gupta [56] who supplemented Protein + carbohydrate + vitamin and minerals and observed a honey area of 9800 cm^2^, as well as Kumari and Kumar [57] who supplemented Bee-sup and documented a honey area of 1963.5 cm^2^. In a study conducted by Mahmood *et al*. [58], they provided colonies with a Pollen supplemental gram diet consisting of 250 g, along with brewer’s yeast and sugar supplements. They observed a maximum honey area of 3820 cm^2^. Another research, carried out by Dogra *et al*. [59], involved the supplementation of various ingredients. The supplemented ingredients included Defatted soya flour (150 g), Wheat flour (150 g), Deactivated yeast (100 g), Sugar (266 g), Water (134 mL), and 40 mL of Rum, making a total of 800 g. They documented a honey area of 602.2 cm^2^.

However, in the current study, a different approach was taken. Kumar and Agrawal [7] conducted an experiment where they fed a mixture of 16.7% defatted soya flour, 16.7% brewery yeast, 8.3% Parched Gram, 8.3% Protein Hydrolysate, 33.3% sugar, and 16.7% glucose. Their findings showed a total colony area of 59.7 cm^2^, which was lower than the results obtained in the present study. Interestingly, the colonies in the current study exhibited exceptional performance, with increased feed intake and higher nutritional value. Additionally, the colonies exhibited a larger brood area, stronger colony strength, and higher nectar content. These factors showed a positive correlation with the honey area. This aligns with the research conducted by Chaand *et al*. [52] and Sihag and Kaur [53], who similarly found that colony strength influences pollen, nectar, and honey area. Furthermore, studies by Tolera [60] highlight the positive impact of pollen supplements on brood area, colony strength, and honey storage.

In the present study, the experimental diets had an impact on the pollen area. The findings of this study revealed that the pollen area (measuring 68.7 cm^2^) was comparatively smaller than the results reported by Sihag and Gupta [56]. In their study, they administered Protein + carbohydrate + vitamin and mineral supplements and observed a maximum pollen area of 252 cm^2^. Similarly, Mahmood *et al*. [58] supplemented the diet with Pollen (250 g per colony) along with brewer’s yeast and sugar, which resulted in a pollen area of 127.9 cm^2^. In a study conducted by Irandoust and Ebadi [61], who examined the effects of wheat gluten on pollination, a pollen area of 143.5 cm^2^ was reported. Another study by Hunde [50], focused on soya bean diets and recorded a pollen area of 219.9 cm^2^. In the present study, colonies that were administered T_4_ and T_2_ treatments demonstrated a higher collection of pollen compared to other treatments. Control colonies, on the other hand, had a lower amount of pollen compared to the colonies under different treatments. This result can be attributed to the variations in their feed intake and the size of their brood area.

According to research conducted by Sihag and Gupta [56] as well as El-Wahab [40], it has been found that there are strong and statistically significant correlations between unsealed brood and sealed brood with pollen in colonies that are provided with pollen substitute diets. Additionally, the strength of a colony has an impact on the amount of pollen, nectar, and honey area, as observed by Chaand [52]. According to Sihag and Kaur [53], there is a positive correlation between pollen stores and both brood and colony strength. Additionally, Mahfouz [62] and Khan *et al*. [63] have noted that supplementing pollen can enhance food storage within the hive, especially during periods when food availability is limited. Recent studies reveal a decline in the pollen area compared to previous findings. This finding supports the discoveries of Huang [54] and Amro *et al*. [55], affirming that honey bees generally show a preference for natural pollen and nectar over artificial supplements and substitute diets. The quality of larval diets directly affects the metabolic function of adult worker bees, whereby diets that are more suitable for their survival lead to a higher metabolic rate per unit of body mass. Differences in the nutritional quality of larvae’s diet could have an impact on the metabolic function of foraging bees, which in turn can affect their efficiency in finding resources. This, in consequence, may affect the accumulation of pollen and nectar reserves crucial for raising brood and surviving the winter, ultimately impacting the overall success of the bee colony [48, 50, 64].

According to Oskay [65], beekeepers often use supplemental feeding as a strategy to support and sustain their honey bee colonies when nectar and pollen sources are scarce in the natural environment or during agricultural cultivation. Tesfaye [66] further emphasized that providing supplementary pollen feeding during periods of limited resources helps maintain the strength of bee colonies within hives and prepares them for the upcoming natural flow of pollen and nectar. Additionally, Chaabane [67] discovered that monitoring the nutritional status of the colony is crucial in ensuring the health of bees and preventing population declines caused by various factors leading to nutritional shortages. The highest honey yield achieved by colonies that received T_4_ was 7 kg, which was slightly lower than the result reported by El-Wahab *et al*. [40]. In their study, they supplemented the colonies with a mixture of 10 g brewery yeast, 1 g bee honey, 8 g Turmeric and Fenugreek powders, 0.5 g A, D, and E vitamins, 45 g powdered sugar, 20 mL orange juice, 10 mL mint oil, and 30 mL sugar syrup, resulting in a honey yield of 3.7 kg.

On the contrary, Irandoust and Ebadi [61] documented a yield of 8.9 kg when using soya bean flour. In a separate study by Sihag and Gupta [56], a yield of 11.3 kg was obtained when colonies were fed on pea flour. Tolera [60] reported a higher yield of 26.8 kg with colonies fed on pea flour. Furthermore, Islam *et al*. [49] achieved a honey yield of 7.5 kg by offering a combination of 30 g of soybean flour, 15 g of Brewer’s yeast, 5 g of honey, 20 g of powdered sugar, 9.5 g of Fenugreek and Turmeric powder, 20 mL of orange juice, 0.5 g of A, D, and E vitamins, as well as 150 mL of sugar syrup.

In a study conducted by Ahmad *et al*. [43], they observed that using sucrose solution as a feed led to a maximum honey yield of 13 kg. Ullah *et al*. [47], also conducted research using sugar cane syrup, a main component of soybean flour, as a substitute for traditional pollen. Their findings indicated a honey yield of 8.7 kg. Similarly, Hunde [50] reported a honey yield of 11.5 kg from bees-fed soya bean diets. All these results were higher than the findings of the current study. The higher honey yields in these studies were attributed to factors such as brood area, nectar and honey storage, and colony strength. The discoveries made by Sihag and Gupta [56] and El-Wahab *et al*. [40] align with our findings. Likewise, we observed strong and noteworthy correlations between the strength of bee colonies and various factors such as unsealed brood/sealed brood and pollen and honey stores. These correlations were evident across multiple studies that examined colonies fed with pollen substitute diets, including research conducted by El-Wahab *et al*. [40], Shehata [68], Saini *et al*. [69], Tawfik *et al*. [70] and Islam *et al*. [67].

Moreover, there was a noteworthy rise in Honey yield among the bee colonies that consumed supplemental diets compared to the control colonies that solely relied on sugar syrup. Research has shown that robust bee colonies can raise more brood and generate a higher quantity of honey in comparison to weaker colonies [71–73]. The observed increase in the nutritional composition and consumption within these colonies in our study directly correlates with higher honey production. This finding aligns with a previous study by [46] which discovered that when the sum of essential amino acid deficiencies in the diet exceeded the content of leucine, it harmed the performance of honey bee colonies and subsequently reduced their honey yield. According to a study by Crone and Grozinger [74], evidence suggests that in honey bees (*Apis mellifera*), the effects of various stressors can be reduced by ensuring proper nutrition. The quality of nutrition becomes crucial, with pollen playing a vital role as it serves as the primary source of protein and lipids in bee diets. This emphasis on high-quality pollen intake is essential for producing more resilient phenotypes in bees.

According to the findings of Nichols and Ricigliano [75], the integration of algae feeds and supplements into beekeeping methods can offer a sustainable solution to enhance the productivity and overall well-being of honey bee colonies. Additionally, Branchiccela *et al*. [76], El-Seedi *et al*. [77], and Hashem *et al*. [78] revealed that various stressors, including pathogens, insecticides, and insufficient diets, negatively impact the immune system of bees, ultimately affecting their health. In our current investigation, we observed that T_4_ and T_2_ showed greater net profit and colony strength. Although T_4_ had slightly higher values compared to T_2_, the difference was not statistically significant. The reason behind these findings can be attributed to the increased attractiveness, nutritional value, and palatability of the feed provided in these particular treatments. Previous research by Saffari *et al*. [79] highlighted the importance of ensuring that the pollen substitute artificial diet contains the necessary elements, texture, and consistency that are appealing and appetizing to honeybees. It must have nutritional values and be free from anti-nutritional factors. Sihag and Gupta [80] also documented that it must be easy to prepare and economically viable for beekeepers. Therefore, a cheap/low-cost and acceptable pollen substitute diet is one of the prime needs of beekeepers to sustain beekeeping.

## 5. Conclusion and recommendations

All experimental diets used as supplementations in the present study affected honey bee colony performance when compared to unfed colonies. The diet presented by T_4_ (nettle infusion syrup and white sorghum powder) and diet presented by T_2_ (bakery yeast and white sorghum and powdered sugar powder) resulted in better value compared with T_3_ (skimmed milk powder and white sorghum, powdered sugar) and T_1_ (*shiro* and *beso* and syrup) as well as control colonies. Regarding profitability, a higher net profit was obtained from T_4_ and T_2_. Even though the difference between T_2_ and T_4_ was statistically insignificant, T4 was numerically higher than T2. As a result, supplementing nettle infusion syrup and sorghum powder is preferable both in its better effect on honey bee colony performance and low cost. Based on this the following recommendations are forwarded. Stinging nettle and sorghum grows in specific areas; it would be beneficial to expand their distribution, and evaluating the impact of this diet on honey quality would also be advantageous.

## Supporting information

S1 Fig Artificial honey comb preparation. (TIF)

S2 Fig Infusion of stinging nettle and its leaf. (TIF)

S1 Table Effect of diet on nectar area. (DOCX)

S2 Table Effect of diet on pollen area. (DOCX)

S3 Table Effect of diet on honey area. (DOCX)

S4 Table Effect of diet on honey yield. (DOCX)

S1 Raw data (XLSX)
