# Supplementary figures and images for: Effect of various supplements on productive performance of honey bees, in the south Wollo Zone, Ethiopia
Source: PLoS One. 2024 May 29;19(5):e0303579. doi: 10.1371/journal.pone.0303579 (PMC11135746; doi:10.1371/journal.pone.0303579)

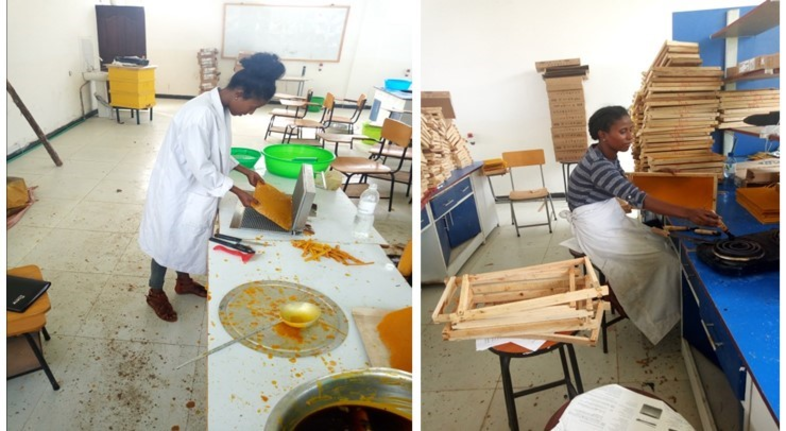

Supplement: S1 Fig — (TIF) [file pone.0303579.s001.tif]

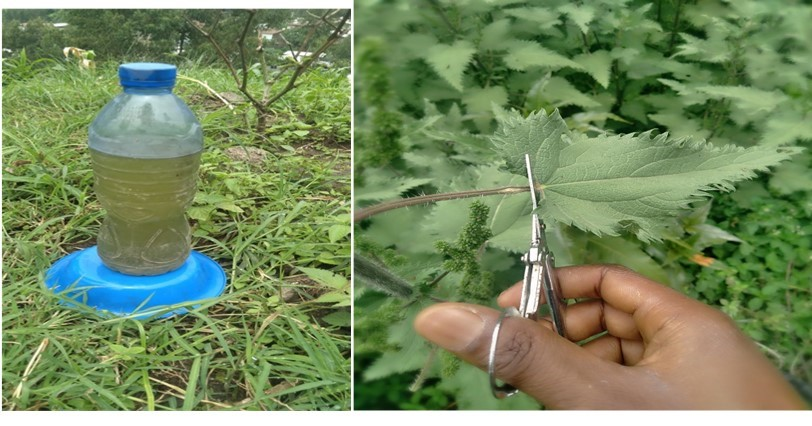

Supplement: S2 Fig — (TIF) [file pone.0303579.s002.tif]
