# Supplementary material for: Effect of various supplements on productive performance of honey bees, in the south Wollo Zone, Ethiopia
Source: PLoS One. 2024 May 29;19(5):e0303579. doi: 10.1371/journal.pone.0303579 (PMC11135746; doi:10.1371/journal.pone.0303579)
Supplement: S1 Table — (DOCX) [file pone.0303579.s003.docx]

**S1:** Effects of different supplemental diets on nectar area (cm^2^) of honey bee colonies on each measurement dates

| **Date** | **T_1_** | **T_2_** | **T_3_** | **T_4_** | **C** | **P value** |
| --- | --- | --- | --- | --- | --- | --- |
| 3_2_2021 | 30.0^a^±1.8 | 30.0^a^±1.8 | 30.0^a^±1.8 | 30.0^a^±1.8 | 30.0^a^±1.8 | 1.0000 |
| 24_2_2021 | 30.2^ba^±1.6 | 34.0^a^±2.6 | 30.8^ba^±1.9 | 34.0^a^±1.9 | 25.0^b^±1.5 | <.0001 |
| 15_3_2021 | 32.2^a^±2.0 | 38.7^a^±2.3 | 34.0^a^±2.7 | 41.2^a^±2.8 | 17.3^b^±0.7 | <.0001 |
| 6_4_2021 | 39.8^b^±3.2 | 57.8^a^±3.5 | 41.8^b^±2.9 | 64.7^a^±4.1 | 7.8^c^±0.5 | <.0001 |
| 27_4_2021 | 48.3^c^±2.3 | 73.7^ba^±3.8 | 58.8^bc^±4.5 | 84.7^a^±5.9 | 4.5^d^±0.4 | <.0001 |
| 28_7_2021 | 30.0^a^±1.9 | 34.0^a^±2.7 | 30.0^a^±1.9 | 34.0^a^±1.9 | 26.5^a^±1.5 | 0.0795 |
| 19_8_2021 | 33.5^b^±2.3 | 42.2^ba^±2.7 | 35.7^ba^±2.6 | 43.5^a^±2.7 | 19.0^c^±1 | <.0001 |
| 10_9_2021 | 42.3^b^±3.1 | 61.7^a^±3.8 | 44.7^b^±3.1 | 66.7^a^±4.0 | 10.8^c^±1 | <.0001 |
| 1_10_2021 | 51.7^b^±3.1 | 78.7^a^±3.8 | 60.8^b^±4.2 | 89.8^a^±5.9 | 6.8^c^±1 | <.0001 |

**T1:** 50% sugar syrup + 14% roasted barley powder (*beso*) + 36% roasted spiced pea powder (*shiro*); **T2:**50% powder sugar + 14% white sorghum powder + 36% bakery yeast; T**3:**50% powder sugar + 14% white sorghum powder + 36% skimmed milk powder **T4:** 50% sugar syrup with infusion of stinging nettle and 1% *kerefa*.+ 50% white sorghum powder; C: not given any supplementation**.** Means with the same letter are not significantly different.
