# Supplementary material for: Effect of various supplements on productive performance of honey bees, in the south Wollo Zone, Ethiopia
Source: PLoS One. 2024 May 29;19(5):e0303579. doi: 10.1371/journal.pone.0303579 (PMC11135746; doi:10.1371/journal.pone.0303579)
Supplement: S2 Table — (DOCX) [file pone.0303579.s004.docx]

**S2:** Effects of different supplemental diets on pollen area (cm^2^) of honey bee colonies on each measurement dates

| Date | T_1_ | T_2_ | T_3_ | T_4_ | C | P value |
| --- | --- | --- | --- | --- | --- | --- |
| 3_2_2021 | 45.0^a^±2.6 | 45.0^a^±2.6 | 45.0^a^±2.6 | 45.0^a^±2.6 | 45.0^a^±2.6 | 1.0000 |
| 24_2_2021 | 35.3^ba^±2.1 | 45.0^a^±2.6 | 40.8^ba^±2.4 | 45.0^a^±2.6 | 30.7^b^±3.8 | 0.0032 |
| 15_3_2021 | 35.3^c^±2.1 | 45.2^ba^±1.8 | 37.2^bc^±1.7 | 50.8^a^±1.9 | 30.3^c^±3.0 | <.0001 |
| 6_4_2021 | 40.3^b^±2.4 | 48.8^ba^±2.6 | 41.2^b^±2.4 | 56.3^a^±3.3 | 14.7^c^±2.2 | <.0001 |
| 27_4_2021 | 46.3^b^±3.2 | 61.7^ba^±5.4 | 51.2^b^±4.9 | 73.2^a^±5.9 | 4.8^c^±0.6 | <.0001 |
| 28_7_2021 | 38.7^a^±0.8 | 43.5^a^±1.3 | 38.8^a^±1.0 | 43.7^a^±1.5 | 31.0^b^±2.9 | 0.0001 |
| 19_8_2021 | 45.5^b^±2.9 | 62.5^a^±4.3 | 56.2^ba^±3.8 | 63.3^a^±4.3 | 23.0^c^±1.5 | <.0001 |
| 10_9_2021 | 62.0^b^±5.5 | 91.3^a^±4.7 | 71.5^b^±5.8 | 95.7^a^±4.8 | 10.2^c^±0.7 | <.0001 |
| 1_10_2021 | 90.5^b^±5.5 | 138.7^a^±7.4 | 97.7^b^±5.1 | 145.0^a^±8.2 | 5.2^c^±0.5 | <.0001 |

**T1:** 50% sugar syrup + 14% roasted barley powder (*beso*) + 36% roasted spiced pea powder (*shiro*); **T2:**50% powder sugar + 14% white sorghum powder + 36% bakery yeast; T**3:**50% powder sugar + 14% white sorghum powder + 36% skimmed milk powder **T4:** 50% sugar syrup with infusion of stinging nettle and 1% *kerefa*.+ 50% white sorghum powder; C: not given any supplementation**.** Means with the same letter are not significantly different.
