# Supplementary material for: Effect of various supplements on productive performance of honey bees, in the south Wollo Zone, Ethiopia
Source: PLoS One. 2024 May 29;19(5):e0303579. doi: 10.1371/journal.pone.0303579 (PMC11135746; doi:10.1371/journal.pone.0303579)
Supplement: S3 Table — (DOCX) [file pone.0303579.s005.docx]

**S3:** Effects of different supplemental diets on honey area (cm^2^) of honey bee colonies on each measurement dates (Mean ±SE)

| Date | T_1_ | T_2_ | T_3_ | T_4_ | C | P value |
| --- | --- | --- | --- | --- | --- | --- |
| 3_2_2021 | 300.0^a^±36.5 | 300.0^a^±36.5 | 300.0^a^±36.5 | 300.0^a^±36.5 | 300.0^a^±36.5 | 1.0000 |
| 24_2_2021 | 228.0^ba^±28.6 | 258.7^ba^±32.4 | 240.3^ba^±29.8 | 264.5^a^±34.7 | 138.2^b^±20.1 | 0.0375 |
| 15_3_2021 | 231.5^a^±28.3 | 271.3^a^±32.5 | 246.0^a^±29.3 | 279.5^a^±34.7 | 75.0^b^±7.8 | 0.0001 |
| 6_4_2021 | 236.7^a^±28.2 | 287.0^a^±32.3 | 251.0^a^±29.2 | 291.0^a^±34.4 | 37.7^b^±3.0 | <.0001 |
| 27_4_2021 | 241.0^a^±27.7 | 305.7^a^±30.5 | 253.0^a^±31.3 | 306.8^a^±33.5 | 21.7^b^±2.7 | <.0001 |
| 28_7_2021 | 238.0^a^±30.6 | 270.8^a^±35.9 | 241.5^a^±35.3 | 280.8^a^±33.7 | 152.2^a^±26.5 | 0.0754 |
| 19_8_2021 | 257.3^ba^±34.5 | 290.7^a^±36.5 | 265.2^a^±32.1 | 308.7^a^±41.4 | 118.3^b^±26.6 | 0.0055 |
| 10_9_2021 | 267.3^a^ ±34.9 | 300.7^a^±20.3 | 277.7^a^±31.2 | 345.0^a^±36.8 | 61.7^b^±10.5 | <.0001 |
| 1_10_2021 | 289.5^b^±38.9 | 375.2^ba^±32.7 | 285.8^b^±31.4 | 424.3^a^±40.1 | 30.0^c^±5.5 | <.0001 |

**T1:** 50% sugar syrup + 14% roasted barley powder (*beso*) + 36% roasted spiced pea powder (*shiro*); **T2:**50% powder sugar + 14% white sorghum powder + 36% bakery yeast; T**3:**50% powder sugar + 14% white sorghum powder + 36% skimmed milk powder **T4:** 50% sugar syrup with infusion of stinging nettle and 1% *kerefa*.+ 50% white sorghum powder; C: not given any supplementation**.** Means with the same letter are not significantly different. SE, Standard error.
