# Supplementary material for: Effect of various supplements on productive performance of honey bees, in the south Wollo Zone, Ethiopia
Source: PLoS One. 2024 May 29;19(5):e0303579. doi: 10.1371/journal.pone.0303579 (PMC11135746; doi:10.1371/journal.pone.0303579)
Supplement: S4 Table — (DOCX) [file pone.0303579.s006.docx]

**S4:** Effects of different supplemental diets on honey yield of honey bees at the end of two experimental periods on each measurement dates

| Date | T_1_ | T_2_ | T_3_ | T_4_ | C | P value |
| --- | --- | --- | --- | --- | --- | --- |
| 28_4_2021 | 2.6^c^±0.3 | 5.0^ba^±0.6 | 3.0^bc^±0.5 | 6.0^a^±0.8 | 1.0^c^±0.2 | <.0001 |
| 2_10_2021 | 3.0^dc^±0.2 | 7.0^ba^±1.1 | 4.7^bc^±0.9 | 8.0^a^±1.0 | 0.3^d^±0.1 | <.0001 |

**T1:** 50% sugar syrup + 14% roasted barley powder (beso) + 36% roasted spiced pea powder (shiro); **T2:**50% powder sugar + 14% white sorghum powder + 36% bakery yeast; T**3:**50% powder sugar + 14% white sorghum powder + 36% skimmed milk powder **T4:** 50% sugar syrup with infusion of stinging nettle and 1% kerefa.+ 50% white sorghum powder; C: not given any supplementation**.** Means with the same letter are not significantly different.
